# TNF Lectin-Like Domain Restores Epithelial Sodium Channel Function in Frameshift Mutants Associated with Pseudohypoaldosteronism Type 1B

**DOI:** 10.3389/fimmu.2017.00601

**Published:** 2017-05-29

**Authors:** Anita Willam, Mohammed Aufy, Susan Tzotzos, Dina El-Malazi, Franziska Poser, Alina Wagner, Birgit Unterköfler, Didja Gurmani, David Martan, Shahid Muhammad Iqbal, Bernhard Fischer, Hendrik Fischer, Helmut Pietschmann, Istvan Czikora, Rudolf Lucas, Rosa Lemmens-Gruber, Waheed Shabbir

**Affiliations:** ^1^Department of Pharmacology and Toxicology, University of Vienna, Vienna, Austria; ^2^APEPTICO GmbH, Vienna, Austria; ^3^Vascular Biology Center, Medical College of Georgia, Augusta University, Augusta, GA, United States

**Keywords:** lectin-like domain of tumor necrosis factor, TIP peptides, solnatide (AP301), amiloride-sensitive epithelial sodium channel, pseudohypoaldosteronism type 1B

## Abstract

Previous *in vitro* studies have indicated that tumor necrosis factor (TNF) activates amiloride-sensitive epithelial sodium channel (ENaC) current through its lectin-like (TIP) domain, since cyclic peptides mimicking the TIP domain (e.g., solnatide), showed ENaC-activating properties. In the current study, the effects of TNF and solnatide on individual ENaC subunits or ENaC carrying mutated glycosylation sites in the α-ENaC subunit were compared, revealing a similar mode of action for TNF and solnatide and corroborating the previous assumption that the lectin-like domain of TNF is the relevant molecular structure for ENaC activation. Accordingly, TNF enhanced ENaC current by increasing open probability of the glycosylated channel, position N511 in the α-ENaC subunit being identified as the most important glycosylation site. TNF significantly increased Na^+^ current through ENaC comprising only the pore forming subunits α or δ, was less active in ENaC comprising only β-subunits, and showed no effect on ENaC comprising γ-subunits. TNF did not increase the membrane abundance of ENaC subunits to the extent observed with solnatide. Since the α-subunit is believed to play a prominent role in the ENaC current activating effect of TNF and TIP, we investigated whether TNF and solnatide can enhance αβγ-ENaC current in α-ENaC loss-of-function frameshift mutants. The efficacy of solnatide has been already proven in pathological conditions involving ENaC in phase II clinical trials. The frameshift mutations αI68fs, αT169fs, αP197fs, αE272fs, αF435fs, αR438fs, αY447fs, αR448fs, αS452fs, and αT482fs have been reported to cause pseudohypoaldosteronism type 1B (PHA1B), a rare, life-threatening, salt-wasting disease, which hitherto has been treated only symptomatically. In a heterologous expression system, all frameshift mutants showed significantly reduced amiloride-sensitive whole-cell current compared to wild type αβγ-ENaC, whereas membrane abundance varied between mutants. Solnatide restored function in α-ENaC frameshift mutants to current density levels of wild type ENaC or higher despite their lacking a binding site for solnatide, previously located to the region between TM2 and the C-terminus of the α-subunit. TNF similarly restored current density to wild type levels in the mutant αR448fs. Activation of βγ-ENaC may contribute to this moderate current enhancement, but whatever the mechanism, experimental data indicate that solnatide could be a new strategy to treat PHA1B.

## Introduction

Tumor necrosis factor (TNF) is a mammalian inflammatory cytokine, which exerts a plethora of effects primarily aimed at defending the host against invading pathogens. Apart from mediating its activities through cross-linking with specific receptors on the surface of mammalian cells (1), TNF participates in innate immune functions through a lectin-like (TIP) domain, spatially distinct from the TNF-receptor binding site (2–4). The lectin-like domain of TNF recognizes and interacts with specific oligosaccharide moieties, in particular *N*,*N*′-diacetylchitobiose (5). TNF is crucially involved in the control of *Trypanosoma brucei brucei* and *T. cruzei* infections, through the trypanolytic effect triggered by interaction of its lectin-like domain with the N-linked *N*,*N*′-diacetylchitobiose core of the variant surface glycoproteins (VSG) of these organisms (1, 2, 6–14). Another effect of the TNF TIP domain observed in early work was the amiloride-sensitive increase in membrane conductance in microvascular endothelial cells (MVECs) (4) and alveolar epithelial cells (15), an effect which we now know is due to activation of the amiloride-sensitive epithelial sodium channel (ENaC) (16). The potential physiological role of the lectin-like domain of TNF in resolution of alveolar edema has been demonstrated in various rodent models of flooded lungs (15, 17, 18). Furthermore, transgenic mice expressing a mutated TNF lectin-like domain are more prone to develop lung edema than their wild-type (WT) counterparts when challenged with the bacterial toxin pneumolysin (PLY) (19).

The synthetic, cyclic, 17-residue peptide, solnatide, mimics the lectin-like domain (TIP) of human TNF (2). Like TNF, TIP peptide can influence regulation of alveolar fluid balance. Solnatide has been shown to activate fluid reabsorption in *in situ* and *in vivo* flooded rat lung models (18) and a mouse version the TIP peptide, mTIP, decreased pulmonary edema in isolated, endotoxin-injured rabbit lung (20). Moreover, solnatide, instilled intratracheally into rats prior to lung transplantation, significantly improved lung function, indicating its use as a potential therapy for ischemia reperfusion injury associated with lung transplantation (21). Inhalation of nebulized solnatide in a porcine bronchoalveolar lavage (BAL) model of acute lung injury (ALI) resulted in an increased PaO_2_/FiO_2_ ratio and reduced extravascular lung water index (EVLWI) (22). More recently, solnatide demonstrated profound therapeutic activity in a rat model of pulmonary edema induced by acute hypobaric hypoxia and exercise (23).

Solnatide activates both endogenously and heterologously expressed ENaC by increasing the open state probability, *P_o_*, of the channel (16, 24, 25). The oligosaccharide-binding property of the TIP domain of TNF plays an important role in the mechanism by which TNF and solnatide interact with and activate ENaC, although the exact nature of this interaction is not yet understood. Elimination of the Na^+^ current-enhancing effect of solnatide following PNGase F-mediated deglycosylation of A549 and H441 cells or of HEK-293 cells heterologously expressing human ENaC suggested that TIP interacts with carbohydrate groups on the extracellular loop of ENaC subunits (16, 19). Proof of the importance of interaction with glycosylated residues in the extracellular loop of ENaC for TIP potentiation of Na^+^ current was obtained from studies with heterologously expressed ENaC in which the five Asn glycosylation sites in the extracellular loop of alpha ENaC had been removed, singly or multiply, by mutation to Gln (26).

The current-potentiating effect of solnatide not only manifests itself in channel kinetics but also in abundance of ENaC subunits at the membrane. We have observed a temporary increase in abundance of α-, β-, γ-, and δ-ENaC 5 and 10 min after prior exposure of HEK-293 cells transiently expressing ENaC to solnatide, but after 1 h, levels return to those seen in the absence of solnatide (16, 26). The solnatide-induced increase in membrane abundance of pore-forming α- and δ-ENaC subunits is significant statistically whereas that of β- and γ-ENaC subunits only slight (26).

In this study, we explore the mechanism of TIP activation of ENaC in electrophysiological and Western blotting experiments using TNF and solnatide. Direct interaction of TNF with ENaC has hitherto not been reported, and so its physiological role in alveolar liquid clearance (ALC) during lung inflammation has been largely inferred from numerous studies with solnatide. A recent study, which sought to determine the precise mechanism by which solnatide stimulated Na^+^ uptake in the presence or absence of PLY, demonstrated that TIP activates ENaC through binding to the carboxyl-terminal domain of the α subunit (19). In the present study, we investigated how native TNF affects Na^+^ current and membrane abundance of ENaC subunits in cells heterologously expressing WT hENaC and mutant hENaC and compared and contrasted these observations to our findings with solnatide.

Surprisingly, solnatide rescues the loss-of-function phenotype in ENaC mutants carrying mutations at conserved positions in α-, β-, and γ-ENaC known to cause pseudohypoaldosteronism type 1B (PHA1B), restoring current levels in these mutant ENaC-expressing cells to WT levels or even higher (27). PHA1B is a very rare inherited disease caused by mutations in the genes encoding the α (SCNN1A), β (SCNN1B), or γ (SCNN1G), subunit of ENaC, resulting in defective transepithelial sodium transport (28). PHA1B usually manifests itself in the neonatal period with life-threatening salt loss, hyperkalemia, acidosis, and elevated aldosterone levels due to end-organ resistance to aldosterone. Patients suffering from PHA1B are at risk from life-threatening, salt-losing crises, combined with severe hyperkalemia and dehydration throughout their entire lives (29, 30). There is as yet no definitive treatment for PHA1B other than supportive management aimed to reduce sodium wasting and hyperkalemia and to restore water–electrolyte and acid–base balance.

In the work reported here, we investigate whether α-ENaC frameshift mutants known to cause PHA1B are also rescued by solnatide, even though they lack the carboxyl-terminal domain of α-ENaC previously postulated to be the site of interaction of solnatide with ENaC (19, 31).

## Materials and Methods

### Cell Culture

Human alveolar epithelial A549 cells (ATCC no. CCL-185) in passages 80–97 and human embryonic kidney HEK-293 cells (ATCC no. CRL-1573) in passages 3–25 were seeded in Dulbecco’s modified Eagle medium/F12 nutrient mixture Ham plus l-glutamine (DMEM/F-12; Gibco^TM^ by Life Technologies, LifeTech Austria), supplemented with 10% fetal bovine serum (FBS; Gibco^TM^ by Life Technologies, LifeTech Austria) and 1% penicillin–streptomycin (Sigma-Aldrich, Vienna, Austria). Cells were maintained at 37°C with 5% CO_2_ in a humidified incubator.

### Molecular Biological Methods

cDNAs encoding α-, β-, and γ-hENaC were a kind gift from Dr. Peter M. Snyder (University of Iowa, Carver College of Medicine, Iowa City, USA). cDNA-encoding δ-hENaC was a kind gift from Dr. Mike Althaus (Justus-Liebig University, Giessen, Germany).

#### Site-Directed Mutagenesis

Point mutations of α-, β-, γ- and δ-hENaC and PHA1B frameshift mutations of α-hENaC were prepared with the QuikChange Lightning Site-Directed Mutagenesis Kit (Agilent Technologies, CA, USA). Mutagenic primers were designed individually with the Primer Design Program provided on the producer’s website or for the frameshift mutations the same base changes as reported in patients (see Table 2) were performed. Primers were ordered from Sigma-Aldrich, Vienna, Austria.

Mutant strand synthesis, digestion of template, and transformation were performed according to the manufacturer’s protocol, and plasmid DNA was extracted from *Escherichia coli* (*E. coli*) cells using the GeneJET Plasmid Miniprep Kit (Thermo Scientific, Loughborough, UK). The mutant DNA was checked by sequencing from LGC Genomics GmbH, Berlin, Germany.

Larger amounts of DNA were provided by amplifying WT or mutant α-, β-, γ-, or δ-hENaC in DH5α competent cells (Invitrogen by Thermo Fisher Scientific, CA, USA) and then extracting DNA using the Plasmid Midi Kit (QIAGEN GmbH, Hilden, Germany).

#### Transfection

HEK-293 cells were transfected 1 day after cell seeding using X-tremeGENE HP DNA transfection reagent (Roche Diagnostics, Mannheim, Germany) according to the manufacturer’s protocol. A set of WT or mutant αβγ- or δβγ-hENaC, or α-, β-, γ-, or δ-hENaC alone was used, and the ratio of DNA to transfection reagent was 1:3. The expression was highest 48–72 h after transfection.

### Cell Surface Biotinylation and Western Blotting

Cell surface biotinylation was performed as previously described (26). In brief, A549 cells or transiently transfected HEK-293 cells were grown in 10 cm dishes in 37°C, 5% CO_2_ incubator in DMEM medium supplemented with 5% FBS. Cells were treated with 40 nM TNF or 200 nM solnatide for 5, 10, or 30 min when 90% confluency had been reached. Medium was aspirated, and then cells were washed twice with 10 ml ice-cold phosphate-buffered saline (PBS), covered with 2.5 mg EZ-Link Sulfo-NHS-SS-Biotin (Thermo Scientific, Rockford, USA), dissolved in 10 ml ice-cold PBS, and incubated at 4°C with gentle agitation for 30 min. Fifty milliliters of quenching solution were added to cells; then cells were scraped in solution and transferred to fresh 50 ml tube. Cell suspension was centrifuged at 500 × *g* for 3 min. Supernatant was discarded, and 5 ml of Tris-buffered saline (TBS) was added to the cell pellet. The cell pellet was resuspended and centrifuged at 500 × *g* for 3 min. Supernatant was discarded and cell pellet was resuspended in lysis buffer containing protease inhibitor cocktail (10 µM pepstatin A, 10 µM phenylmethylsulfonyl fluoride, and 10 µM leupeptin) and transferred to fresh 1.5 ml centrifuge tube. Cell pellet was then homogenized on ice by ultrasonication using 1 s bursts and incubated on ice for at least 30 min. Intact cells and nuclei were pelleted by centrifugation at 10,000 × *g* for 2 min under cooling conditions. Pellet was then discarded and supernatant was transferred to fresh tube, incubated overnight with 0.5 ml NeutrAvidin Agarose under gentle rotation at 4°C and centrifuged at 500 × *g* for 5 min under cooling conditions. Supernatant was then discarded and the pellet washed twice with 200 µl lysis buffer. The biotinylated proteins were eluted with 100 µl sodium dodecyl sulfate (SDS) sample buffer (62.5 mM Tris, pH 6.8, 1% SDS, 10% glycerine, 50 mM dithiothreitol) containing 10 µM E64 at 65°C for 10 min. Sample was then centrifuged at 500 × *g* for 2 min. Pellet was discarded and supernatant subjected to protein electrophoresis and immunoblotting. The biotinylated proteins were separated under reducing conditions by SDS-PAGE using 7.5% SDS gel along with prestained protein marker (cat. #12949 from Cell Signaling). Proteins were then transferred onto a nitrocellulose membrane (UltraCruzTM 0.45 mm, Santa Cruz Biotechnology, TX, USA) by semi-dry blotting at 25 V for 30 min. Unspecific binding sites were blocked by incubating the membrane overnight at 4°C with 3% FBS in PBS supplemented with 0.02% sodium azide. Membrane was then incubated for 90 min with primary antibody (anti-α-hENaC, anti-δ-hENaC, and anti-β-actin from Sigma Aldrich; anti-β- and anti-γ-hENaC from Santa Cruz Biotechnology). Membrane was washed 5× with 10 ml PBS containing 0.1% Tween-20 (PBST), and corresponding horseradish peroxidase-conjugated secondary antibodies (Santa Cruz Biotechnology) were applied. After 90-min incubation, membrane was washed 3× with PBST and once with PBS. Enhanced chemiluminescence (ECL) substrate (Amersham ECL Plus Western Blotting Detection Reagent, GE Healthcare, Vienna, Austria) was used for visualization. Following incubation for 2 min, membranes were exposed to X-ray films (Amersham Hyperfilm ECL, GE Healthcare). Exposed films were scanned and quantified using ImageJ (NIH, MD, USA).

### Electrophysiology

Electrophysiological experiments were performed as described in detail by Shabbir et al. (16). Briefly, effects of TNF and solnatide on WT and mutated hENaC were studied on transfected HEK-293 cells at room temperature (19–22°C) 24–48 h after plating. Currents were recorded with the patch clamp method in the whole-cell mode. The chamber contained 1 ml of the bath solution of the following composition (in mM): 145 NaCl, 2.7 KCl, 1.8 CaCl_2_, 2 MgCl_2_, 5.5 glucose, and 10 HEPES, adjusted to pH 7.4 with 1 M NaOH solution. Micropipettes were pulled from thin-walled borosilicate glass capillaries (Harvard Apparatus, Holliston, MA, USA) with a DMZ Zeitz Puller to obtain electrode resistances ranging from 2 to 5 MΩ. The pipette solution contained (in millimolars): 135 potassium methane sulfonate, 10 KCl, 6 NaCl, 1 Mg_2_ATP, 2 Na_3_ATP, 10 HEPES, and 0.5 EGTA, adjusted to pH 7.2 with 1 M KOH solution. Chemicals for pipette and bathing solutions were supplied by Sigma-Aldrich (Vienna, Austria). Electrophysiological measurements were carried out with an Axopatch 200B patch clamp amplifier (Axon Instruments, CA, USA). Capacity transients were canceled, and series resistance was compensated. Whole-cell currents were filtered at 5 kHz and sampled at 10 kHz. Data acquisition and storage were processed directly to a PC equipped with pCLAMP 10.2 software (Axon Instruments, CA, USA). After GΩ-seal formation, the equilibration period of 5 min was followed by control recordings at a holding potential of –100 mV. Then, aliquots of a stock solution, which was prepared with distilled water, were cumulatively added into the bath solution. The wash-in phase lasted about 1–5 min. After steady-state had been reached, the same experimental protocol was applied for each concentration of TNF and solnatide as well as during control recordings.

### Statistical Analysis

Data were analyzed with OriginPro 2017 (OriginLab, Northampton, MA, USA) and figures were edited with CorelDRAW X7 (Corel Corporation, Ottawa, ON, Canada). Data are represented as mean ± SEM of at least three independent biological replicates/experiments. Significant differences of two independent values were evaluated by unpaired Student’s *t*-test. Whereas one-way ANOVA followed by Tukey’s *post hoc* test was used when groups of data were compared with each other. The type of statistical test is indicated in the figure legends. In case no specific test is mentioned, ANOVA was performed.

### Test Compounds

Tumor necrosis factor (CAS Registry Number 94948-59-1, Sigma-Aldrich, Austria) and the TNF lectin-like domain derived peptide solnatide, also known as AP301 and called TIP peptide [CAS Registry Number: 259206-53-6; CA Index Name: l-cysteine, l-cysteinylglycyl-l-glutaminyl-l-arginyl-l-.alpha.-glutamyl-l-threonyl-l-prolyl-l-.alpha.-glutamylglycyl-l-alanyl-l-.alpha.-glutamyl-l-alanyl-l-lysyl-l-prolyl-l-tryptophyl-l-tyrosyl-, cyclic (1.fwdarw.17)-disulfide], with the amino acid sequence CGQRETPEGAEAKPWYC were tested for their ability to activate wild-type and mutant ENaC. Synthesis and description of solnatide is reported in detail by Hazemi et al. (24).

## Results

### Electrophysiological TNF–ENaC Interaction

In a recent study by Czikora et al. (19) the authors postulated for the first time a direct interaction between the cytokine TNF and the amiloride-sensitive sodium ion channel in a multiple step manner, starting with the interaction with glycosylated membrane components, followed by caveolae-dependent uptake and finally binding to the carboxyl-terminal domain of the α-subunit. This mode of action would suggest a physiological role of the lectin-like domain of TNF in ALC. However, in Czikora’s study, using a cyclic peptide which mimics the lectin-like domain of TNF, it was not demonstrated directly that native TNF can also activate ENaC *via* these proposed mechanisms.

**Figure 1:**
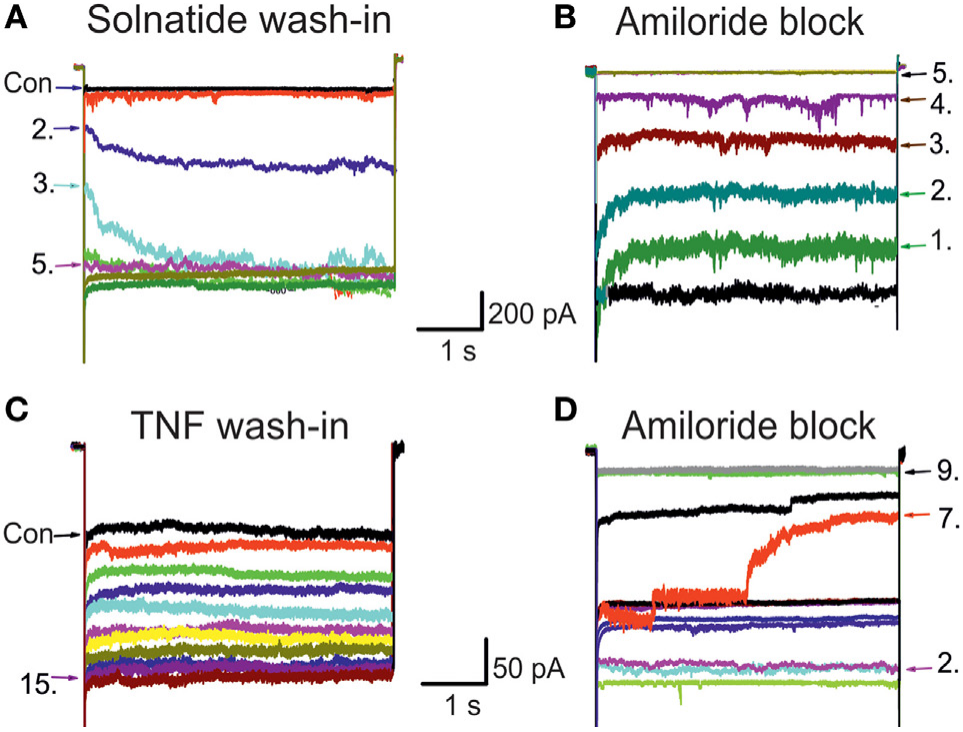
**Original traces of wild-type (WT) epithelial sodium channel (ENaC) showing solnatide and tumor necrosis factor (TNF) wash-in and amiloride block**. The control whole-cell current (Con; untreated) of HEK-293 cells transiently transfected with WT αβγ-ENaC. Each set of traces represents current measured when cells were clamped at –100 mV, using 20 s pulse intervals. Pulse numbers are indicated to show the time course of wash-in and amiloride block. **(A)** Typical 200 nM solnatide wash-in; 5th pulse showed the steady-state level. **(B)** Typical 10 µM amiloride block of 200 nM solnatide-induced current; 5th pulse showed full block of inward sodium current. **(C)** Typical 20 nM TNF wash-in; 15th pulse showed the steady-state level. **(D)** Typical 10 µM amiloride block of 20 nM TNF-induced current; 9th pulse showed full block of inward sodium current.

In previous experiments with A549 cells that endogenously express α-, β-, γ-, and δ-subunits, we could demonstrate a current activating effect by both TNF as well as the TIP peptide solnatide (24), and this increase in current by TNF (Figure 1) and solnatide (16) was confirmed in heterologously expressed αβγ-ENaC and individual ENaC subunits. The onset of action was slower with TNF compared to solnatide and was blocked by 10 µM amiloride within a few pulses (Figure 1). Therefore, as a next step, we studied the effect of native TNF on hENaC in more detail.

To study single subunits and mutant ENaC HEK-293 cells were used, as no mRNA encoding ENaC subunits has been found in untransfected HEK-293 cells indicating no endogenous expression of ENaC (40). Only cells with a clear amiloride response and with significantly higher current than non-transfected (16) and mock-transfected (27) HEK-293 cells were used for data analysis. In αβγ-ENaC heterologously expressed in HEK-293 cells, TNF enhanced amiloride-sensitive sodium current with approximately 8-fold higher potency (EC_50_: 6.7 ± 2.1 nM) than solnatide (EC_50_: 54.7 ± 2.2 nM), and TNF was even about 13-fold more effective than solnatide in α- and δ-ENaC subunits (Table 1). Notably, however, the maximal steady-state current level of the TNF-activated current in αβγ-ENaC was significantly (*p* < 0.001, *n* = 7) lower than that of solnatide-induced current (Table 1). The main targets of TNF were the pore-forming subunits α- and δ-ENaC, similar to what has been shown previously for solnatide (16) (Table 1). Interestingly, compared to solnatide, the current-activating effect of TNF was more pronounced in the individual subunits, but without statistically significant difference. The weakest current increase by TNF was found in the γ-subunit, so that no reliable EC_50_ value could be estimated.

**Table 1:** **Comparison of the effect of tumor necrosis factor (TNF) and solnatide on amiloride-sensitive Na^+^ current**.

hENaC subunit(s)	Amiloride-sensitive control current (pA)	Maximal induced current (pA)		EC_50_ (nM)	
		Tumor necrosis factor (TNF) (*n* = 5)	Solnatide^a^	TNF (*n* = 5)	Solnatide^a^
αβγ	75.8 ± *4.5*	162.5 ± 7.5***	953.2 ± *11.5*	6.7 ± 2.1***	54.7 ± *2.2*
α	55.3 ± *5.5*	48.1 ± 5.0***	11.3 ± *6.2*	4.2 ± 1.9***	57.8 ± *3.4*
β	11.5 ± *3.7*	18.5 ± *5.5*	n.d.	18.8 ± *2.9*	n.d.
γ	14.0 ± *5.1*	8.0 ± *3.3*	n.d.	n.d.	n.d.
δ	60.6 ± *2.5*	27.8 ± 2.1***	15.6 ± *7.9*	5.0 ± 0.3***	63.5 ± *9.9*

*^a^Data from Ref. (16)*.

***p < 0.001, t-test, significant difference between maximal TNF- and solnatide-induced current and significant difference between EC_50_ values for TNF and solnatide, respectively.

*n.d., no detectable current*.

*Values are given as mean ± S.E*.

In A549 cells single channel open probability, mean open time, number and duration of bursts were significantly increased by TNF and solnatide without affecting conductivity of the channel, and this increase was completely abolished in PNGase F pretreated cells (16). To verify which of the putative *N*-linked glycosylation sites participate in binding of TNF to the extracellular loop we generated single N (asparagine) to Q (glutamine) mutants in the human α-subunit at each potential glycosylation site (N232, N293, N312, N397, N511) and co-expressed these mutated α-subunits in HEK-293 cells along with βγ-subunits. Similar to solnatide, but less pronounced, we could show that each *N*-glycan was involved in TNF-induced increase in current with position αN511 being the most important glycosylation site. For comparison, the maximal TNF-induced current of 162.5 ± 7.5 pA in αβγ-ENaC (*n* = 7) was significantly lower in αN232Qβγ-ENaC with 84.7 ± 8.8 pA (*p* < 0.001, *n* = 5), and attenuation of TNF-induced current was most pronounced in the mutant αN511Qβγ-ENaC with 50.7 ± 5.3 pA (*p* < 0.001, *n* = 4).

### TNF and Membrane Abundance of ENaC

**Figure 2:**
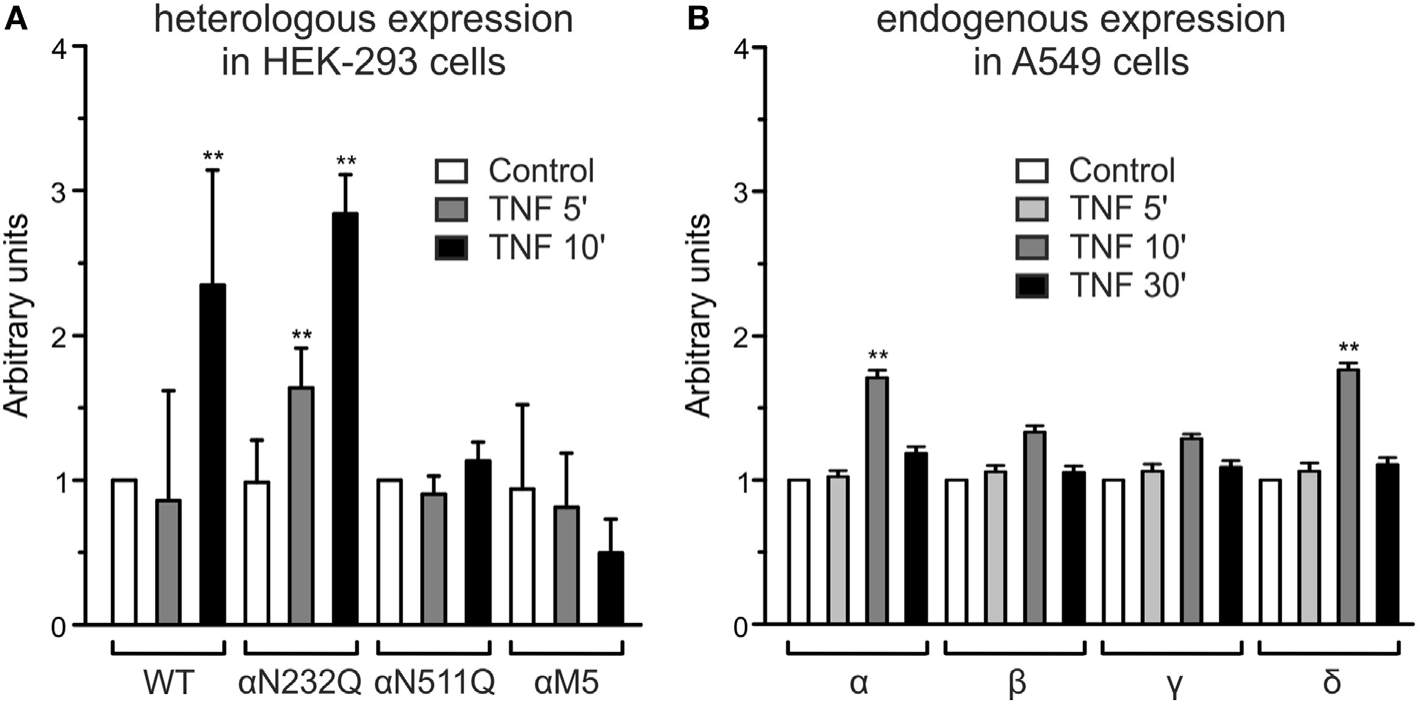
**Effect of tumor necrosis factor (TNF) on membrane abundance of N-linked glycosylation site mutations in the extracellular loop of α-epithelial sodium channel (ENaC) and of single subunits of ENaC**. **(A)** A complex of wild-type (WT) αβγ- or single αN232Q and αN511Q mutants as well as quintuple α-ENaC mutant (N232Q, N293Q, N312Q, N397Q, N511Q) combined with WT βγ-ENaC was heterologously expressed in HEK-293 cells, untreated (control) or treated with 40 nM TNF for 5 or 10 min. Biotinylated surface proteins were analyzed using Western blot; the expression of α-ENaC was normalized compared to β-actin and set in relation to WT control (=1). Significant differences are indicated, **p < 0.01 (*n* = 3). **(B)** Biotinylated surface proteins from A549 cells untreated or after 5, 10, or 30 min treatment with 40 nM TNF, heterologously expressing αβγδ-ENaC, were analyzed with anti-α-, β-, γ-, or δ-ENaC antibodies. The expression was normalized to β-actin and set in relation to its respective control. Significant differences are indicated, **p < 0.01 (*n* = 3).

**Figure 3:**
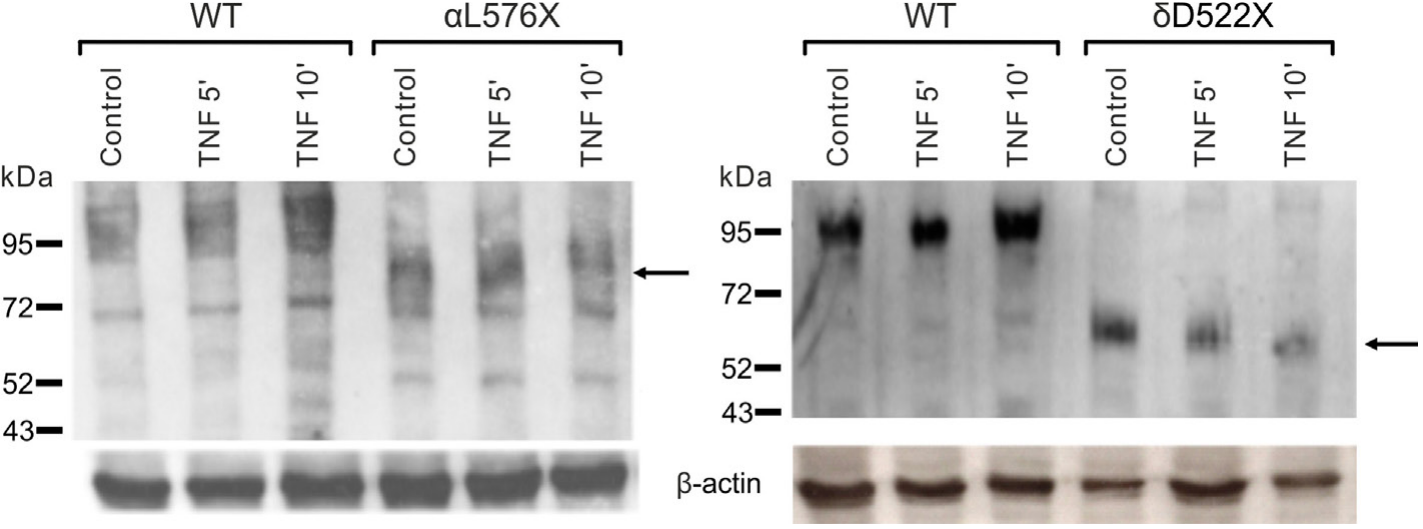
**Effect of tumor necrosis factor (TNF) on membrane abundance of αL576X and δD522X mutants**. Mutant αL576X (left blot) or δD522X (right blot) was co-expressed with wild-type (WT) βγ-hENaC in HEK-293 cells. WT αβγ- or δβγ-epithelial sodium channel (ENaC) was used as reference. Cells were treated with 40 nM TNF for 5 or 10 min, as indicated, or untreated (control). Biotinylated surface proteins were blotted and visualized with anti-α-ENaC (left blot) or anti-δ-ENaC (right blot) antibodies. WT α- and δ-ENaC show a band at about 95 kDa, whereas the truncated mutants are shorter (the relevant bands are indicated by arrows). A representative blot out of three independent biological replicates is shown in each case.

In A549 cells (Figure 2B), as well as in heterologously expressed α- and δ-ENaC, treatment with 40 nM TNF caused a significant, transient increase in membrane abundance of α- and δ-subunits after 10 min (*p* < 0.01, *n* = 4), while the increase of the β- and γ-subunit was not significant. The increased expression of α- and δ-subunits returned to control values after 30 min (Figure 2B). These results confirm data obtained in presence of solnatide (26) with the only difference that for TNF a longer incubation time of 10 min was needed to observe an increase in membrane expression of α- and δ-subunits. Furthermore, our data with TNF on the membrane expression level also underline the importance of the *N*-linked glycosylation sites for the interaction of the cytokine with the ion channel. WT and single or quintuple (αM5) α-subunit mutants (N232Q, N293Q, N312Q, N397Q, N511Q) were transfected along with βγ-hENaC in HEK-293 cells. Expression levels of αN232Q mutant in presence of TNF were comparable to WT, whereas in αN511Q and α-ENaC lacking all five glycosylation sites an increase of membrane abundance of α-ENaC by 40 nM TNF was inhibited (Figure 2A). These results indicate that in the α-subunit, position N511 plays a prominent role in the interaction of TNF with ENaC (Figure 2A). As Czikora et al. (19) postulated that the carboxyl terminal of α-hENaC is essential for the interaction with the lectin-like domain of TNF, we deleted the carboxyl-terminal domain by introducing stop codons at L576 in α-hENaC to generate αL576X, as well as in the δ-subunit at position D522 to create δD522X. In these mutants, no increase in membrane abundance could be observed in presence of TNF (Figure 3), which again confirms the data with solnatide (26).

### α-ENaC Frameshift Mutations

Unexpectedly, solnatide rescues the loss-of-function phenotype in ENaC mutants (27) carrying mutations at conserved positions in α-, β-, and γ-ENaC known to cause PHA1B. Since the α-subunit is supposed to play a prominent role in the ENaC current activation by TNF and TIP peptide, we investigated whether TNF and solnatide can also enhance αβγ-ENaC current in α-ENaC loss-of-function frameshift mutants, i.e., αI68fs, αT169fs, αP197fs, αE272fs, αF435fs, αR438fs, αY447fs, αR448fs, αS452fs, and αT482fs (Table 2), which have been reported to cause PHA1B. These frameshift mutants lack the carboxyl-terminal domain of α-ENaC previously postulated to be the site of interaction of solnatide with ENaC (19, 31). Apart from αI68fs all studied frameshift mutants originate in the extracellular loop of α-ENaC predominantly clustering in the thumb. Worth mentioning, all described frameshift mutations have the WT sequence before the mutation and some random amino acids after the mutations, until a stop codon occurs. The theoretical total length of the truncated proteins is indicated in Table 2.

**Table 2:** **Total protein length and affected regions of α-frameshift mutations that are verified to occur in PHA1B patients**.

Mutant (protein)	Truncated protein length (AA)	Affected region	Domain location in homology model of mouse α-ENaC (32)	Mutation in patient (DNA)	First published in
αI68fs	142	Exon 2 cytoplasmic	Intracellular	203delTC	(33)
αT169fs	203	Exon 3 extracellular loop	Finger	505delAC	(34)
αP197fs	204	Exon 3 extracellular loop	Finger	587-588insC	(35)
αE272fs	309	Exon 4 extracellular loop	Finger	814-815insG	(36)
αF435fs	480	Exon 8 extracellular loop	Thumb	1305delC	(34)
αR438fs	480	Exon 8 extracellular loop	Thumb	1311delG	(36)
αY447fs	458	Exon 8 extracellular loop	Thumb	1340insT	(37)
αR448fs	459	Exon 8 extracellular loop	Thumb	1342-1343insTACA	(35)
αS452fs	480	Exon 8 extracellular loop	Thumb	1356delC	(38)
αT482fs	495	Exon 10 extracellular loop	Palm	1449delC	(39)

#### Solnatide Restores Amiloride-Sensitive Sodium Current in Frameshift Mutations of α-ENaC

**Figure 4:**
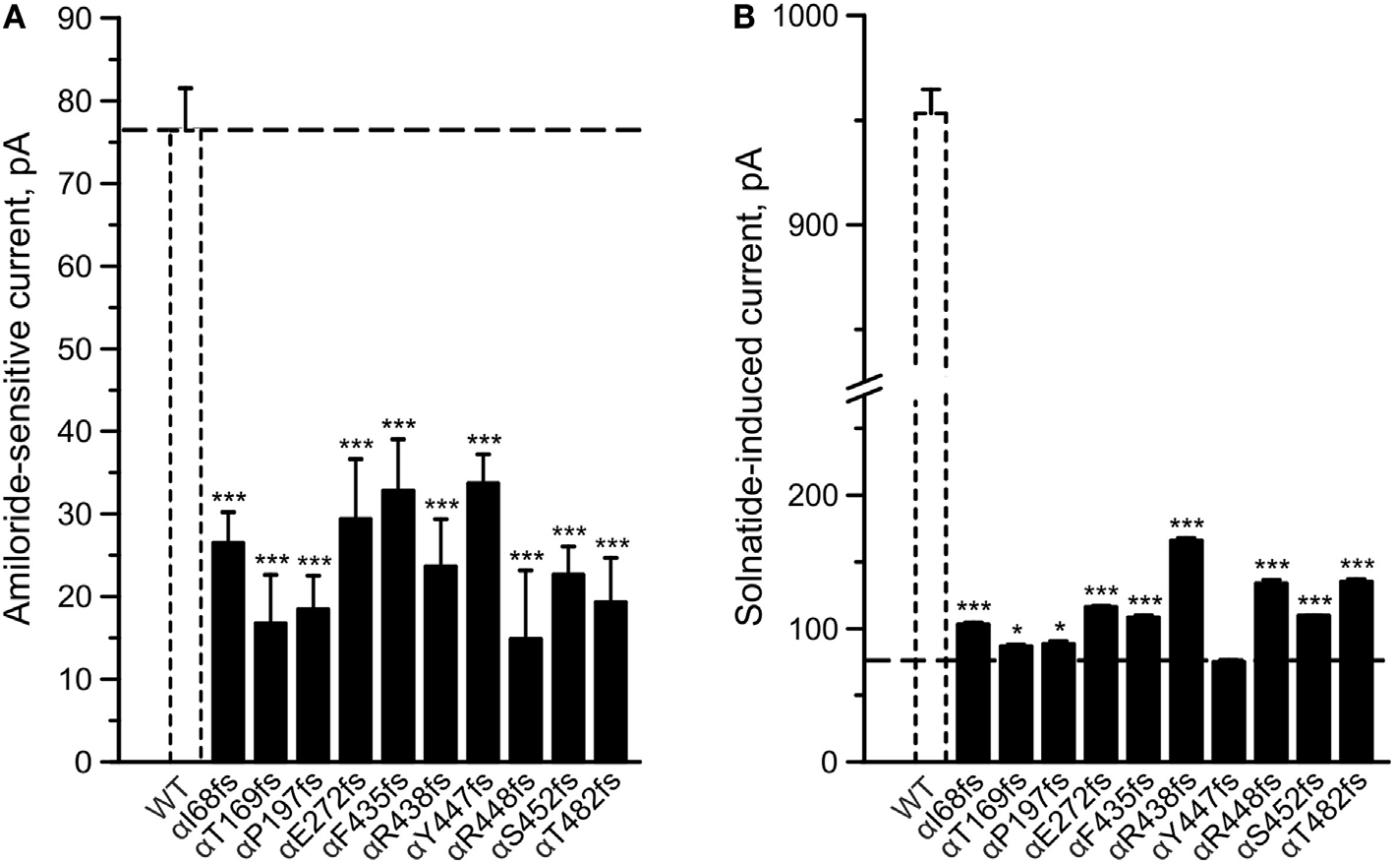
**Amiloride-sensitive sodium current and solnatide-induced current in pseudohypoaldosteronism type 1B (PHA1B) frameshift mutants**. Wild-type (WT) or mutant α-epithelial sodium channel (ENaC) was co-expressed with βγ subunits in HEK-293 cells. Cells were patched in the whole-cell mode, and the inward current was elicited at −100 mV. The 10 µM amiloride-sensitive current **(A)** and 200 nM solnatide-induced current **(B)** of 10 frameshift mutations in α-ENaC associated with PHA1B (black bars) are shown in relation to WT (white, broken bar). For comparison the amiloride-sensitive current of WT αβγ-ENaC is indicated as broken line. Significant difference compared to WT control was calculated using one-way ANOVA followed by Tukey’s *post hoc* test, *p < 0.05, ***p < 0.001 (*n* = 3–11).

**Figure 5:**
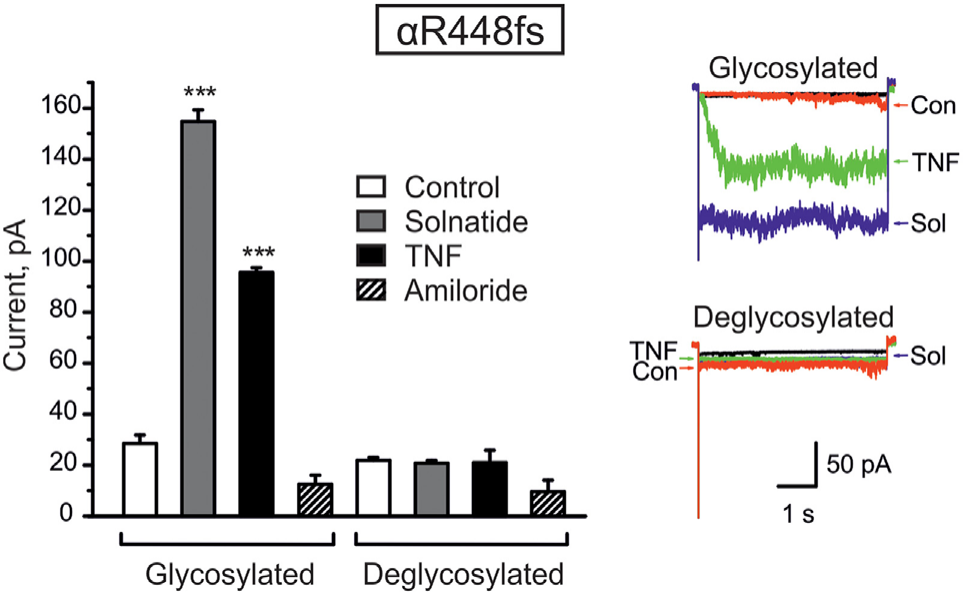
**Deglycosylation of R448fs with PNGase F abolished both solnatide- and tumor necrosis factor (TNF)-induced activation**. Mean values of 200 nM solnatide- and 20 nM TNF-induced inward currents in control (glycosylated) and 100 units PNGase F treated (deglycosylated) αR448fsβγ (left), ***p < 0.001 compared with control as determined by unpaired Student’s *t*-test, *n* = 3. Typical solnatide- and TNF-induced current traces of αR448fsβγ in control and PNGase F (100 U) treated transiently transfected HEK-293 cells. For comparison, original traces from separate solnatide and TNF experiments are superimposed (right).

To determine whether TNF and solnatide not only activate Na^+^ current in WT αβγ-ENaC but also in PHA1B-causing α-ENaC frameshift mutations, experiments were performed by transfecting mutant α-ENaC together with WT βγ-subunits into HEK-293 cells. The macroscopic amiloride-sensitive Na^+^ currents of all investigated α-ENaC frameshift PHA1B mutants were significantly (*p* < 0.001, for number of experiments see Table 3) decreased compared to WT control level (Figure 4A). Remarkably, solnatide was able to activate the reduced current in all studied frameshift mutants up to or even higher than WT control current in absence of solnatide (Figure 4B), even though these mutants lack the carboxyl-terminal domain of α-ENaC previously postulated to be the site of interaction of solnatide with ENaC (19, 31). A maximum level of concentration-dependent current activation was reached at 200 nM with EC_50_ values as indicated in Table 3. TNF was also able to activate current in mutant ENaC; in case of αR448fs (Figure 5) up to WT control current without treatment (compare with Figure 4). Similar to WT ENaC the maximal TNF-induced current is lower than the solnatide-induced current in αR448fs mutant (compare Figure 5 and Table 1), but the approximately 3.5-fold increase (TNF-induced/amiloride-sensitive current) in mutant ENaC exceeds the 2-fold current activation in WT ENaC after treatment with TNF. Mutant solnatide (T6A, E8A, E11A), which had no current-activating effect on WT ENaC (24), also had no effect on PHA1B mutants αF435fs and αR448fs.

**Table 3:** **EC_50_ values of pseudohypoaldosteronism type 1B frameshift mutants for solnatide**.

Construct	EC_50_	*n*
Wild-type (WT)	54.7 ± 2.2	11
αI68fs	73.4 ± 13.4	9
αT169fs	58.3 ± 5.2	9
αP197fs	84.0 ± 4.9***	5
αE272fs	75.6 ± 5.4***	5
αF435fs	64.6 ± 8.7	7
αR438fs	56.6 ± 8.0	7
αY447fs	57.2 ± 4.8	7
αR448fs	50.8 ± 2.4	3
αS452fs	68.5 ± 4.1**	5
αT482fs	50.8 ± 5.5	5

Significant difference compared to WT was calculated with the unpaired Student’s t-test, **p < 0.01, ***p < 0.001.

#### Varied Effect of Solnatide on Membrane Abundance of α-ENaC Frameshift Mutants

To study α-ENaC protein abundance in plasma membrane cell surface, biotinylation of HEK-293 cells transiently transfected with WT or different PHA1B mutants was followed by SDS-PAGE and immunoblotting. Expression of frameshift mutants varied markedly. For example, expression of αF435fs, αY447fs, αR448fs, and αT482fs was highly significantly (*p* < 0.001, *n* = 4), and αP197fs was significantly (*p* < 0.01, *n* = 4) increased compared to WT. Notably, expression of the two mutants αF435fs and αT482fs was strikingly increased although the amiloride-sensitive Na^+^ current was significantly (*p* < 0.001, *n* = 7 and *p* < 0.001, *n* = 5, respectively) attenuated (Figure 4A). Expression of αR438fs and αS452fs was comparable to WT ENaC, whereas expression of αI68fs, αT169fs, and αE272fs was significantly (*p* < 0.001, *n* = 4) decreased compared to expression of WT ENaC. Treatment of HEK-293 cells expressing WT or mutant frameshift α-ENaC with solnatide led to a transient and significant increase in membrane abundance of α-ENaC (Table 4).

**Table 4:** **Effect of solnatide on membrane abundance of α-epithelial sodium channel frameshift mutations**.

Mutation	Control	Solnatide	
		5 min	10 min
Wild-type	1	1.48 ± 0.12***	1.40 ± 0.09***
αI68fs	0.15 ± 0.06	0.25 ± 0.07*	0.32 ± 0.11**
αT169fs	0.54 ± 0.06	0.80 ± 0.03***	0.46 ± 0.09
αP197fs	1.18 ± 0.05	1.33 ± 0.07**	0.48 ± 0.09***
αE272fs	0.35 ± 0.06	0.72 ± 0.03***	0.64 ± 0.05***
αF435fs	2.37 ± 0.15	3.71 ± 0.20***	2.82 ± 0.17**
αR438fs	0.90 ± 0.07	1.39 ± 0.14***	0.87 ± 0.10
αY447fs	1.27 ± 0.10	1.57 ± 0.13***	1.89 ± 0.16***
αR448fs	1.32 ± 0.10	5.88 ± 0.32***	1.30 ± 0.12
αS452fs	1.11 ± 0.25	1.36 ± 0.11***	1.94 ± 0.08***
αT482fs	4.67 ± 0.27	6.23 ± 0.24***	4.58 ± 0.30

*p < 0.05, **p < 0.01, ***p < 0.001, significant difference from respective control values (one-way ANOVA, Tukey’s *post hoc* test).

#### Deglycosylation of α-ENaC Frameshift Mutations

We have previously shown that glycosylation of the extracellular loop of ENaC is one of the prerequisites of solnatide-induced ENaC activation (26). To validate the role of glycosylation in TNF- and solnatide-induced amiloride-sensitive Na^+^ current activation in frameshift mutants, cell surface expression and patch-clamp experiments were performed following PNGase F treatment of αR448fsβγ as an example. As shown in Figure 5, no current could be induced by TNF or solnatide in αR448fs (*n* = 3) mutants when preincubated with PNGase F.

Taken together, these results indicate that frameshift mutation αR448fsβγ requires glycosylation of extracellular sites of ENaC for solnatide and TNF-induced activation of amiloride-sensitive sodium current.

**Figure 6:**
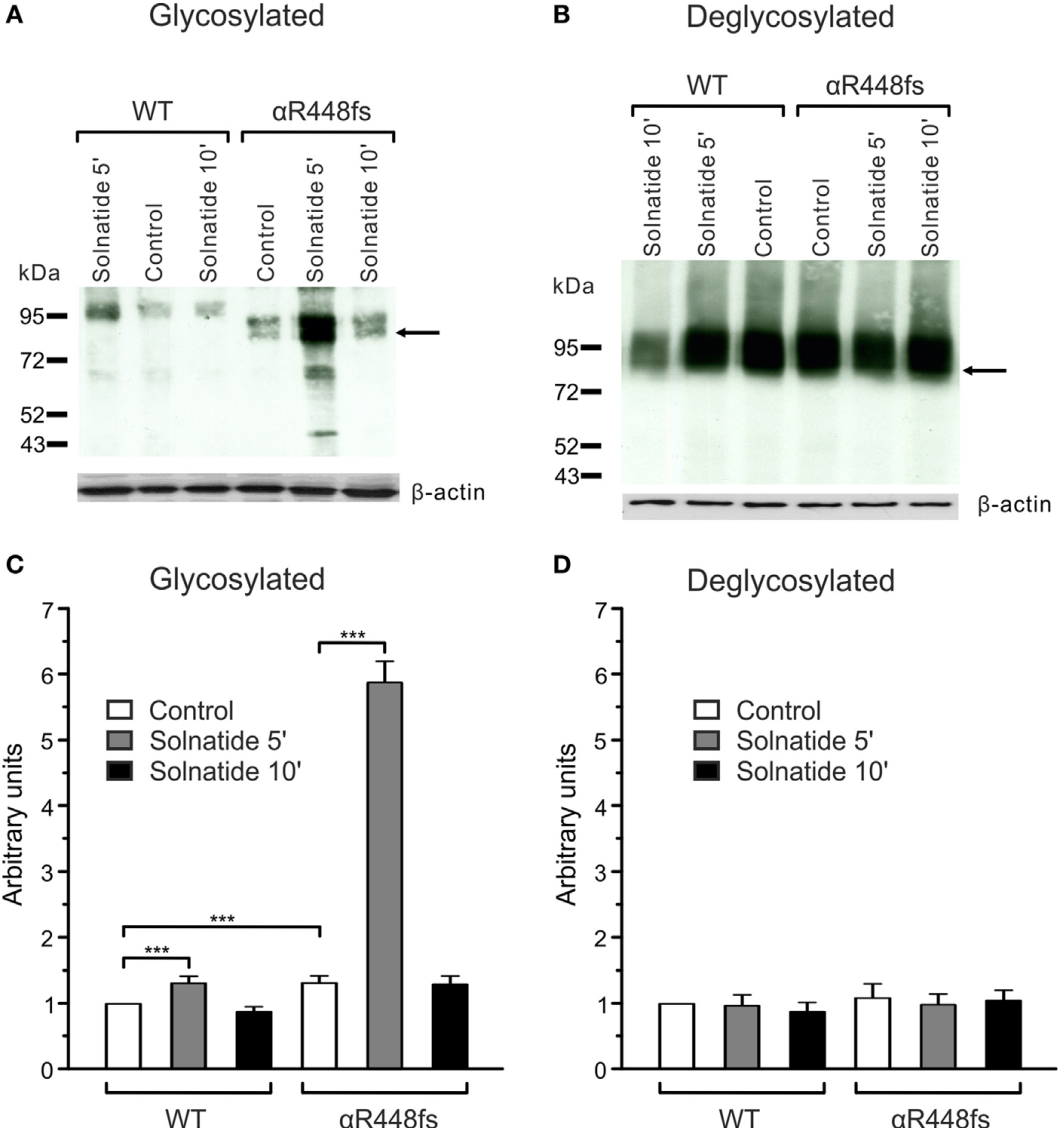
**Effect of solnatide on the membrane abundance of αR448fs without and with PNGase F treatment**. Biotinylated surface proteins of HEK-293 cells heterologously expressing WT αβγ-epithelial sodium channel (ENaC) or αR448fsβγ-ENaC treated with 200 nM solnatide at indicated time points and/or 100 units PNGase F were blotted and analyzed with anti-α-ENaC antibody. One representative blot out of four independent biological replicates is shown before [**(A)**; glycosylated] and after PNGase F treatment [**(B)**; deglycosylated]. Wild-type (WT) α-ENaC shows a band at about 95 kDa and for mutant α-ENaC the protein band, which was used for quantification is indicated by arrows. α-ENaC expression was normalized to β-actin and set in relation to WT control (= 1). The membrane abundance of glycosylated αR448fs-ENaC is highly increased after 5 min of solnatide treatment **(C)**, whereas after PNGase F treatment no differences can be observed [**(D)**; deglycosylated]. Significant differences are indicated, ***p < 0.001 (*n* = 4).

**Figure 7:**
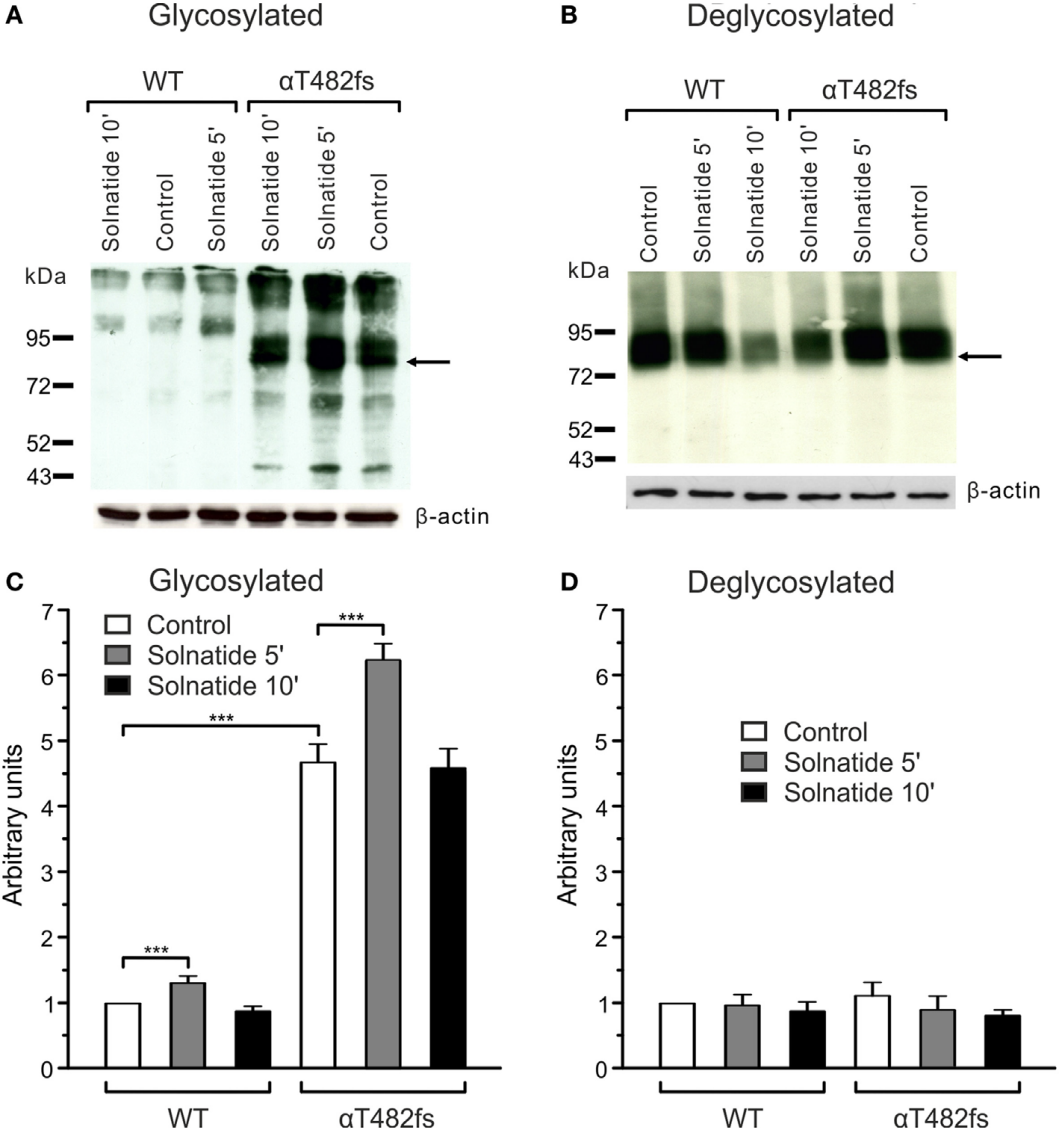
**Effect of solnatide on the membrane abundance of αT482fs without and with PNGase F treatment**. Biotinylated surface proteins of HEK-293 cells heterologously expressing wild-type (WT) αβγ-epithelial sodium channel (ENaC) or αT482fsβγ-ENaC treated with 200 nM solnatide at indicated time points and/or 100 units PNGase F were blotted and analyzed with anti-α-ENaC antibody. One representative blot out of four independent biological replicates is shown before [**(A)**; glycosylated] and after PNGase F treatment [**(B)**; deglycosylated]. WT α-ENaC shows a band at about 95 kDa and for mutant α-ENaC the protein band which was used for quantification is indicated by arrows. α-ENaC expression was normalized to β-actin and set in relation to WT control (=1). The membrane abundance of glycosylated αT482fs-ENaC is already increased without solnatide (control) compared to WT and even more after 5 min of solnatide treatment **(C)**, whereas after PNGase F treatment no differences can be observed [**(D)**; deglycosylated]. Significant differences are indicated, ***p < 0.001 (*n* = 4).

For studies on the role of glycosylation in expression, the two mutants, αR448fs and αT482fs, were chosen, because they showed a marked increase in membrane expression in presence of solnatide. As illustrated in Figures 6 and 7, the solnatide induced increase in membrane abundance was completely abolished in deglycosylated mutants.

#### Role of β- and γ-ENaC in α-ENaC Frameshift Mutations

Lucas et al. (31) identified positions V567, E568, and E571 in the α-subunit as the crucial sites for binding of the lectin-like domain of TNF. They generated alanine replacement mutants in this region of α-ENaC and examined its interaction with the TIP peptide. In triple V567A/E568A/E571A and double V567A/E568A mutants, they found reduced binding capacity of the TIP peptide. Despite the absence of these relevant binding sites in our studied α-frameshift mutants, solnatide caused a significant current increase. As solnatide showed a small current increase in WT βγ-ENaC, we created alanine mutants (βM2γM2) in equivalent residues of β(E539A, E542A)- and γ(E548A, E551A)-ENaC. E539 and E542 in β-ENaC and E548 and E551 in γ-ENaC are homologous to E568 and E571 in the α-subunit, whereas the V567 residue of α-ENaC is I538 in β-ENaC and I547 in γ-ENaC. Solnatide, however, still increased the amiloride-sensitive sodium current in these mutants, which implies that these regions in β- and γ-ENaC do not play any role in the current-activating effect of α-frameshift mutations.

## Discussion

We have previously shown that the synthetic cyclic peptide solnatide, which mimics the lectin-like domain of TNF, requires one of the two pore-forming α- and δ-ENaC subunits to induce its maximum amiloride-sensitive sodium current-activating effect (16). Loss-of-function mutations in ENaC genes translate into the salt-wasting genetic disease PHA1B (33, 41). We have also shown that loss-of-function point mutations of ENaC found in PHA1B patients conduct significantly low current when transfected along with WT β- and γ-subunits (27). Remarkably, amiloride-sensitive currents were restored to WT control levels by solnatide and its congener, AP318 (27). In the present study, experiments were performed to elucidate the effect of the TNF lectin-like domain, both as an integral part of the TNF molecule as well as represented by solnatide, on PHA1B frameshift mutations.

### Lectin-Mediated Activation of ENaC by TNF

The mechanism of TNF-induced ion channel modulation has been intensively studied and, in particular, TNF in combination with other cytokines could drive a pathological condition to a more aggressive state (42, 43). However, as a possible therapeutic molecule, the machinery of TNF-induced activation of ion channels is still largely unknown (44). TNF exhibits a dual role of action in pathological conditions; specifically, TNF has been shown to contribute to the pathogenesis and development of pulmonary edema, through binding to TNF receptors and consequent initiation of the inflammatory cascade. However, some studies have demonstrated surprisingly that TNF can also promote alveolar fluid reabsorption *in vivo* and *in vitro*, a protective effect mediated by the lectin-like domain of the cytokine, which is spatially distinct from the TNF-receptor binding sites (45).

The current-enhancing effect of TNF on different ion channels including ENaC has been documented (15, 46). We have previously shown that solnatide, mimicking the lectin-like domain of TNF, can activate WT ENaC channels (16, 24, 26), as well as ENaC carrying PHA1B-causing mutations (27). In the present study, our data provide evidence for a mechanism of lectin-like domain-mediated, TNF-induced activation of ENaC carrying PHA1B-causing frameshift mutations. Our data demonstrate that TNF has to bind to ENaC glycosylation sites of the extracellular loop through its lectin-like domain in order to exert its ENaC-activating effect as well as to increase translocation of newly synthesized channels to the plasma membrane. Notably, one TNF molecule, which exists as a stable homotrimer (47) contains three lectin-like domains which make the TNF a highly potent activator of ENaC compared to solnatide (a single lectin-like domain mimicking molecule); see EC_50_ values in Table 1. In contrast, the maximal stimulatory effect of solnatide was greater and was reached more rapidly after ~2-min exposure, compared to that of TNF, which was reached after ~5 min (Figure 1). The maximum induced current for solnatide was 953.2 ± 11.5 pA compared to 162.5 ± 7.5 pA induced by TNF (Table 1). The reason for slower time course of activation and smaller current induced by TNF could be that TNF is a bulkier, larger molecule (the mature TNF trimer has a molecular mass of approximately 52 kD) than solnatide (17-mer cyclic peptide, molecular mass 1.9 kD) and hence occupies more space around the extracellular loop of ENaC, around which in comparison more molecules of solnatide, could be accommodated and simultaneously engage with glycosylation or other sites of interaction.

An alternative interpretation of slower TNF time course of activation of ENaC compared to solnatide could be that TNF and solnatide interact with both the extracellular and intracellular domains of ENaC; the time required to activate ENaC would simply reflect the necessity of TNF and solnatide to penetrate the plasma membrane. Binding of the triad of lectin-like domains at the tip of the native TNF homotrimer to glycosylation sites on the ENaC heterooligomer might hinder further folding and subsequent penetration of the TNF molecule across the plasma membrane. We have previously shown that solnatide required αβγ-ENaC or δβγ-ENaC to show its maximum stimulatory effect (16). To our surprise, in single subunit experiments, TNF-induced current was higher than solnatide-induced current (Table 1).

Direct interaction of TNF with ENaC has hitherto not been reported and so its physiological role in improving ALC during lung inflammation has been largely inferred from numerous studies with solnatide and other TIP peptides (18, 20–23). A recent study which sought to determine the precise mechanism by which solnatide stimulated Na^+^ uptake in the presence or absence of PLY, demonstrated that TIP activates ENaC through binding to the carboxyl-terminal domain of the α-subunit (19). Using heterologously expressed WT ENaC we show in the present study that native TNF enhances Na^+^ current, although the maximum TNF-activated current is less than with solnatide.

Tumor necrosis factor, like solnatide, also requires the intracellular carboxyl-terminal region of α- or δ-ENaC to exert its effect of bringing about an increase in membrane abundance of the respective subunits (Figure 3). Thus, with the mutants αL576Xβγ-ENaC and δD522Xβγ-ENaC, which lack the region between TM2 and the carboxyl terminus of α- or δ-hENaC, respectively, the increase in membrane abundance seen with WT α- or δ-ENaC was not observed (Figure 3). These data are in agreement with our previously published reports (19, 26, 31) that the carboxyl-terminal domain of α- or δ-ENaC is an essential motif for TNF lectin-like domain induced activation of the channel.

The possibility exists that TNF binds to the cell membrane surrounding or in the vicinity of oligomeric ENaC in a general, non-specific manner, thereby altering the disposition of ENaC in the bilayer and resulting in a conformation with a higher *P_o_*. Such an affect would still be amiloride-sensitive if the amiloride-binding sites in ENaC subunits were intact and accessible and would be eliminated by addition of amiloride. A precedent for such non-specific membrane insertion of TNF at low pH has been documented (48). Specifically, a role has been suggested for residues in the lectin-like domain of TNF in membrane insertion. The lectin-like domain occupies residues Cys101-Glu116 of human TNF (2), located in triplicate in the highly flexible loop region at the apex of the bell-shaped native TNF trimer (49). Trp114, the first-ordered residue after the apical flexible loop (47), is buried at pH 7.4 but could readily become exposed to an aqueous milieu upon protonation of nearby residues (e.g., Glu116, a salt-bridge participant), resulting in increased surface hydrophobicity and a tendency for insertion into the lipid bilayer by hydrophobic interactions (48). Moreover, membrane penetration has been shown to stabilize the low pH conformation of TNF, and membrane inserted TNF exhibits a native trimeric structure (48). The corresponding bulky, hydrophobic region of TIP peptides (Trp15 in solnatide) has been shown to be one of the essential characteristic features required for the Na^+^ current-potentiating effect of these peptides (24). In the case of solnatide, the cyclic peptide is totally exposed to the aqueous environment leading one to question whether Trp15 would similarly lend to the peptide the tendency for membrane insertion by hydrophobic interaction. Earlier studies with artificial lysosomes, however, could produce no evidence of direct membrane interaction of this TIP peptide (50), leading the authors to conclude that interaction of the TIP domain *via* an ion channel or other membrane protein was required for its current-potentiating effect.

### Restoration of ENaC Current in Frameshift Mutants by TNF and Solnatide

Previously it has been shown that frameshift mutations in pacemaker channels (*HCN4*) do produce a functional channel, which shows normal intracellular trafficking and membrane integration, when transfected in mammalian cells (51), whereas in the case of the cardiac sodium channel (*SCN5A*), a complete loss-of-function phenotype was reported (52).

Computational and site-directed mutagenesis approaches have shown that the lectin-like domain of TNF and solnatide, the synthetic peptide which mimics it, exert their ENaC-activating effect through binding with glycosylation sites of extracellular loops of ENaC (26, 53). Because solnatide has been shown to directly bind with glycosylation sites of ENaC, we treated αR448fsβγ-ENaC with PNGase F prior to testing in a patch clamp assay with solnatide and TNF. Convincingly, neither TNF nor solnatide potentiated amiloride-sensitive current in αR448fsβγ-ENaC following PNGase F treatment, contrary to the activation observed without prior PNGase F treatment (Figure 5). PNGase F treatment also abolished the increase in membrane abundance observed with mutants αR448fs and αT482fs in the presence of solnatide (Figures 6 and 7). These results indicate and are consistent with our previous results that glycosylation sites on the extracellular loop of ENaC are essential for solnatide-induced activation of ENaC (16, 26).

All the frameshift mutants described in the current work lack the amiloride-binding site of the α-subunit, located in TM2, but retain amiloride-binding sites in the co-expressed WT β- and γ-subunits. The amiloride-binding site of α-ENaC is at S556, the position corresponding to Gly439 in ASIC1 (54) and located in the middle of TM2. Amiloride-binding sites occur at equivalent positions in the β- and γ-ENaC subunits (54, 55). The amiloride sensitivity shown by the PHA1B frameshift mutants described in the present work must therefore be due to amiloride binding to sites in the β- and γ-subunits.

In the present study we analyzed frameshift mutations of α-ENaC which produce a truncated ENaC α-subunit. We found that these frameshift mutants can generate amiloride-sensitive current, but it is significantly lower than WT ENaC (Figure 4A). Remarkably, solnatide restored the amiloride-sensitive current in all these frameshift mutants to WT or higher levels (Figure 4B). As shown in Table 2, these mutants generate a truncated α-ENaC of different lengths ranging from 142 (I68fs) to 495 (T482fs) amino acid residues. The αI68fs mutation results in production of a truncated α-subunit comprising a polypeptide chain of 142 amino acid residues, of which residues 1–67 are WT and 68–142 are non-native due to the shift in the reading frame of the mRNA transcript by two nucleotide positions. Analysis of the 142 mutant amino acid sequence with the TMPred bioinformatics tool for prediction of membrane-spanning regions, failed to detect any TM regions, whereas in WT α-hENaC, TM1 is located between residues F86-F110 by sequence comparison with the ENaC homolog, ASIC1 (56). The 142-residue polypeptide resulting from the αI68fs mutation is unlikely to penetrate the membrane, but may associate intracellularly with β- and γ-subunits and thus be detectable in the biotinylated membrane protein fraction.

Previous work of others had shown that the PHA1B mutant αI68fs conducts 0.1% current compared with WT when co-expressed with rat βγ-ENaC in *Xenopus* oocytes (57). Surprisingly, solnatide induced a current increase in αI68fsβγ-ENaC, which lacks both TM regions and all hitherto known or hypothesized binding motives for solnatide activation, namely: glycosylation sites in the extracellular loop (26), carboxyl-terminal domain of α-ENaC (19); V567 and E568 in TM2, residues found to be critical for solnatide and TNF binding (31).

**Figure 8:**
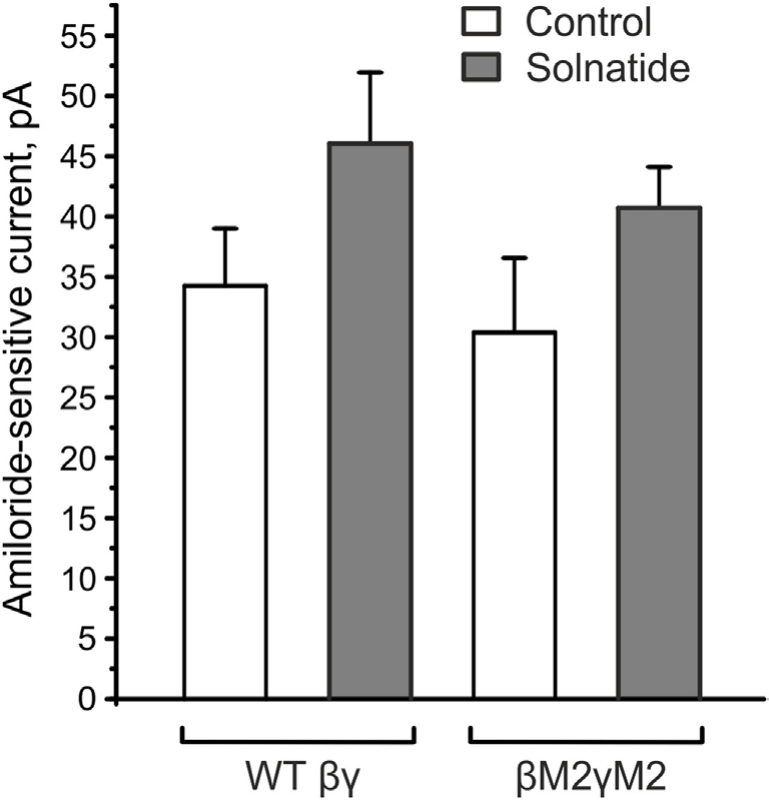
**βE539AE542AγE548AE551A mutants (βM2γM2) did not affect the solnatide-induced activation of βγ-epithelial sodium channel (ENaC)**. HEK-293 cells transiently transfected with wild-type (WT) βγ- or mutant βM2γM2-ENaC were patched in the whole-cell mode. The inward current at −100 mV was measured in absence (control) and presence of 200 nM solnatide and the 10 µM amiloride-sensitive current was calculated. E539 and E542 in β-ENaC and E548 and E551 in γ-ENaC are homologous to the postulated solnatide-binding sites (V567), E568 and E571 in α-ENaC (31), but solnatide was still able to activate βM2γM2-ENaC (not significant) to an equal extent as WT βγ-ENaC (*n* = 3).

The solnatide-induced activation of αI68fsβγ-ENaC could be in part due to the presence of βγ-ENaC subunits co-transfected with mutant αI68fsENaC. In fact, this applies to all the frameshift mutants examined here. To solve this puzzling discrepancy, we analyzed solnatide activation of ENaC comprising the β- and γ-subunits only. As shown in Figure 8, solnatide could activate the inward sodium current through βγ-ENaC channels to a level comparable to that observed for αI68fsβγ-ENaC (Figure 4B). These data indicate and are in agreement with our previously published results (16), namely that solnatide can activate βγ-ENaC marginally. Solnatide has been shown to activate ENaC by binding critical residues located in TM2 of α-hENaC (31). Lucas et al. (31) found that double (V567A,E568A) and triple (V567A, E568A, E571A) α-ENaC mutants showed reduced binding capacity to solnatide and TNF, resulting in an abolition of the increase in *P_o_* usually observed with WT ENaC in the presence of solnatide, although membrane expression was the same as WT. To explore the possibility that the observed potentiation of Na^+^ current in the αI68fsβγ-ENaC mutant could be due to binding of solnatide or TNF to residues of βγ-ENaC equivalent to E568 and E571, two of the three residues in TM2 of α-ENaC studied by Lucas et al. (31), we generated point mutations of β-ENaC: E539A, E542A and γ-ENaC: E548, E551 (Figure 8). A small increase in the amiloride-sensitive Na^+^ current was still observed with βE539A, E542A, γE548A, E551A-ENaC in the presence of solnatide (Figure 8). These results indicate that some other mechanism is responsible for solnatide-induced potentiation of the Na^+^ current, albeit small, in these βγ-ENaC TM2 mutants, which lack α-ENaC and therefore the crucial residues V567A, E568A in TM2, as well as residues in β- and γ-ENaC, E539, E542 and E548, E551, respectively, equivalent to E568 and E571 in α-ENaC. Such a mechanism could explain the Na^+^ current-potentiating effect of solnatide on the αI68fsβγ-ENaC and the other frameshift mutants examined in the present study.

Langloh et al. (58) also studied the effects of mutating residues in TM2 of α-hENaC, but unlike the alanine mutants described above, they mutated highly conserved glutamic acid residues to arginine, E568R, E571R, and D575R, thus reversing the charge at these important positions in TM2. Whole-cell amiloride-sensitive current recorded from oocytes injected with the α-ENaC mutants along with WT β- and γ-ENaC, was low compared with the WT channel, but plasma membrane abundance of the mutant channels was the same as that of WT. The mutations decreased channel conductance but did not affect Na^+^:K^+^ permeability.

Results of earlier experiments conducted by our group with mutants αL576Xβγ-ENaC and δD522Xβγ-ENaC, which lack the region between TM2 and the carboxyl terminus of α- or δ-hENaC (26), had indicated a residual albeit non-significant increase in the amiloride-sensitive current in the presence of solnatide. A channel lacking all carboxyl termini, namely αL576XβD546XγD556X, showed an even slighter, non-significant increase of current after treatment with solnatide in preliminary experiments. This small increase was not seen in the case of αL576Xβγ-ENaC and δD522Xβγ-ENaC in which the glycosylation sites in the extracellular loop had been removed by mutation (26). Thus, apart from the requirement for an intact carboxyl-terminal region in the α-subunit, some other unknown glycosylation-mediated mechanisms seem to play a minor role in TIP activation of ENaC.

A striking feature of the frameshift mutations examined in this work is their non-random distribution in the 3D molecular structure of the α-ENaC subunit. Specifically, of the 10 reported mutations in the current report, five (50%) are located in the thumb domain (Table 2) according to the domain nomenclature established for the ENaC homolog, ASIC1 (56). Of the remaining mutations, three are located in the finger domain, one in the palm domain, and one intracellularly. Another α-ENaC frameshift mutation causing PHA1B previously investigated by our group, S243fs (27, 39), is located in the finger domain. Although we cannot purport to have investigated all known PHA1B mutations (some of which have not been reported in the literature), there does seem to be a trend for the thumb domain of α-ENaC to manifest only frameshift mutations, since all PHA1B-causing mutations so far located to the thumb domain of α-ENaC are frameshift mutations (unpublished findings) translated from exon 8 of the mRNA transcript. All frameshift mutations result in truncated polypeptide chains that contain the α-ENaC amino terminal native sequence preceding the mutation followed by a sequence of non-native residues of varying length, depending on the position of the mutation and length of the out-of-frame mRNA before a stop codon is encountered (Table 2). At the gene level, a mutational hotspot resulting in insertion or deletion of nucleotide base pairs might be the cause of such clustering of mutations in exon 8. The results presented here suggest that at the protein level, since α-subunits are detected by surface biotinylation, a channel with severely reduced Na^+^ conducting capacity is produced, apparently comprised of truncated α-subunit and full-length wild-type β- and γ-subunits. Alternatively, truncated polypeptide chains are trafficked to the membrane, but Na^+^ conducting channels, albeit of severely compromised activity, are assembled from β- and γ-subunits only.

The effect of solnatide on increasing membrane abundance in the frameshift mutants was extremely varied and no trend could be discerned, other than that the effect is transient with a peak around 5−10 min of exposure to solnatide, suggesting that solnatide exerts its effect by increasing trafficking of mutant α-ENaC to the membrane. Some mutants, specifically αF435fs and αT482fs, were characterized by a markedly increased membrane abundance of the truncated subunit compared to WT ENaC in the absence of solnatide, the membrane abundance increasing even further following exposure to solnatide (Table 4). All frameshift mutants described here lack the “PPxY” and “YXXΦ” motifs located in the intracellular carboxyl-terminal region and required for ubiquitination and endocytosis (59, 60), and in the absence of which, mutant subunits would accumulate at the cell surface. This could explain the significantly higher abundance in the membrane of some of the frameshift mutants compared to WT ENaC. The increase in abundance of mutant subunits compared to WT does not seem to correlate with higher amiloride-sensitive current either without or in the presence of solnatide (Figure 4, Table 4), suggesting that these mutant subunits are mostly dysfunctional proteins. Nevertheless, mutant subunits do increase in abundance in response to solnatide and this effect, combined with the increase in *P_o_* brought about by the lectin-like domain interacting with mutant α-subunits and possibly with WT β- and γ-subunits results in solnatide rescuing these PHA1B frameshift mutants and restoring amiloride-sensitive Na^+^ current to physiological levels.

### Concluding Remarks

The results presented here validate the use of TIP peptides as experimental models for the TNF lectin-like domain, previously assumed in numerous studies (19, 21, 31, 45, 61). Although the PHA1B frameshift mutants investigated in the present study lack features shown in earlier studies to be critical for TNF lectin-like domain interaction with ENaC, the fact that solnatide potentiates amiloride-sensitive Na^+^ current to physiological levels, rescuing the mutants, indicates that some additional glycosylation-dependent mechanism, possibly involving β- and γ-ENaC, contributes to the solnatide-induced amiloride-sensitive Na^+^ current. Consequently, as we previously reported for point mutations causing PHA1B (27), TIP peptides would seem to be good candidates for lead compounds in the drug development process for treatment of this life-threatening hereditary disease caused by loss-of-function mutations in ENaC.

## Author Contributions

AW gave substantial contribution to the design of the work, performed experiments, analyzed and interpreted data, and drafted the work. MA performed experiments, analyzed and interpreted data, and drafted the work. ST gave substantial contributions to the conception and design of the work, interpretation of data, and drafted the work. DEM, FP, and SI performed electrophysiological experiments, analyzed data, and drafted the work. ALW, BU, DG, and DM performed Western blot experiments, analyzed data, and drafted the work. BF, HF, and HP gave contribution to the conception of the work and revised it critically. IC and RL interpreted data and revised the work critically for important intellectual content. RL-G gave substantial contribution to the conception and design of the work, interpretation of data, and drafted the work. WS gave substantial contribution to the design of the work, performed experiments, analyzed data, and drafted the work. All the authors approved the version to be published and agree to be accountable for the content of the work.

## Conflict of Interest Statement

The authors declare that this study was funded by a Wellcome Trust Pathfinder Award and the Wirtschaftsagentur Wien Call FemPower 2015. These programs required co-funding by a commercial company. APEPTICO Forschung und Entwicklung GmbH (1150 Vienna, Austria) provided the test compounds, but was not involved in the study design or collection, analysis or interpretation of the data. BF, HF, HP, and ST were employed by APEPTICO Forschung und Entwicklung GmbH (1150 Vienna, Austria). AW and WS were partly financed by APEPTICO Forschung und Entwicklung GmbH, based on the R&D funds received from the Wellcome Trust and the Wirtschaftsagentur Wien. All other authors declare no competing interests.
